# Validity of Physician Billing Claims to Identify Deceased Organ Donors in Large Healthcare Databases

**DOI:** 10.1371/journal.pone.0070825

**Published:** 2013-08-14

**Authors:** Alvin Ho-ting Li, S. Joseph Kim, Jagadish Rangrej, Damon C. Scales, Salimah Shariff, Donald A. Redelmeier, Greg Knoll, Ann Young, Amit X. Garg

**Affiliations:** 1 Division of Nephrology, Department of Medicine, Western University, London, Ontario, Canada; 2 Department of Epidemiology & Biostatistics, Western University, London, Ontario, Canada; 3 Institute for Clinical Evaluative Sciences, Ontario, Canada; 4 Department of Medicine, University of Toronto, Toronto, Ontario, Canada; 5 Department of Critical Care Medicine, Sunnybrook Health Sciences Centre, Toronto, Ontario, Canada; 6 Interdepartmental Division of Critical Care Medicine, University of Toronto, Toronto, Ontario, Canada; 7 Department of Clinical Epidemiology and Biostatistics, McMaster University, Hamilton, Ontario, Canada; 8 Division of Nephrology, University of Ottawa, Ottawa, Ontario, Canada; University of Colorado, United States of America

## Abstract

**Objective:**

We evaluated the validity of physician billing claims to identify deceased organ donors in large provincial healthcare databases.

**Methods:**

We conducted a population-based retrospective validation study of all deceased donors in Ontario, Canada from 2006 to 2011 (n = 988). We included all registered deaths during the same period (n = 458,074). Our main outcome measures included sensitivity, specificity, positive predictive value, and negative predictive value of various algorithms consisting of physician billing claims to identify deceased organ donors and organ-specific donors compared to a reference standard of medical chart abstraction.

**Results:**

The best performing algorithm consisted of any one of 10 different physician billing claims. This algorithm had a sensitivity of 75.4% (95% CI: 72.6% to 78.0%) and a positive predictive value of 77.4% (95% CI: 74.7% to 80.0%) for the identification of deceased organ donors. As expected, specificity and negative predictive value were near 100%. The number of organ donors identified by the algorithm each year was similar to the expected value, and this included the pre-validation period (1991 to 2005). Algorithms to identify organ–specific donors performed poorly (e.g. sensitivity ranged from 0% for small intestine to 67% for heart; positive predictive values ranged from 0% for small intestine to 37% for heart).

**Interpretation:**

Primary data abstraction to identify deceased organ donors should be used whenever possible, particularly for the detection of organ-specific donations. The limitations of physician billing claims should be considered whenever they are used.

## Introduction

Canada is experiencing a major shortfall in deceased organ donations similar to many other countries [1]. In 2011, 3361 Canadians were waiting for a solid organ transplant while 304 patients died waiting [2]. Several initiatives are underway in many countries to increase organ donation including registration of intent to donate, presumed consent policies, financial incentives for donors, and relaxed criteria for donation [3], [4].

Health administrative data contain information collected passively from government and healthcare providers for managing patients [5]. However, they were not designed for research purposes and prone to data entry errors [6]. In addition, there may also be no financial incentive for physicians to provide accurate diagnostic data for billing information [7]. Previous database research has shown that some diagnostic codes and physician claims can be used reliably to identify patients with a certain condition [7], [8], but not others [9]–[11].

Many organ procurement organizations keep detailed records of their activities that require laborious manual chart reviews at multiple hospitals [12]. Obtaining this information from large healthcare administrative databases may be a cost-effective approach and may even provide additional data elements. Further, administrative databases can often be linked with other datasets to provide additional data elements. We conducted a detailed PubMed search with keywords “organ donor”, “organ donation” and “validation” retrieved no prior studies in any jurisdiction describing the validity of algorithms to identify deceased organ donors within administrative healthcare databases.

With respect to data requirements in this field, what is needed is accurate data on: 1) all deaths (data which does exist in many jurisdictions), 2) all deaths eligible for deceased organ donation, 3) all eligible deaths where the family was approached for deceased organ donation, 4) those who died where organs were procured for transplantation (and which organs), and 5) those who died and had organ procured and successfully transplanted into a recipient (and which organs/number of organs per donor). In this study we sought to determine whether administrative databases can be a reliable source to identify step 5) individuals who became a deceased organ donor and those organs transplanted into a recipient.

To identify such donors and the organs transplanted we tested different physician billing algorithms in the provincial administrative databases of Ontario, Canada. We hypothesized that physician billing claims are a reliable source of data to identify deceased donors because organ procurement is a clearly defined procedure. Thus, the presence of a physician billing claim associated with organ procurement should serve as a valid marker for whether the person was an organ donor. The ultimate goal was to determine whether these algorithms can be reliably used in future policy-making, health services delivery planning, quality improvement and research.

## Methods

### Study Design and Setting

We conducted a population-based retrospective validation study testing algorithms to detect deceased organ donors in provincial health administrative databases in Ontario, Canada. Specifically, we compared the performance of a physician billing claims database in identifying deceased donors with a reference standard. Ontario has an estimated population of 13 million [13] and all residents are covered by the province's universal health plan. We next created a cohort of all deaths in Ontario from January 2006 to March 2011 using provincial healthcare administrative databases. The reference standard to determine who was a deceased donor came from a manual review of hospital charts, identified using the records of Ontario's organ procurement organization (Trillium Gift of Life Network, TGLN). We used a standard diagnostic test assessment framework to calculate the sensitivities, specificities, positive predictive values and negative predictive values of algorithms to compare the physician billing database codes to the reference standard. Databases were linked together using unique health card numbers, gender and date of birth.

### Ethics Statement

We conducted our study according to a pre-specified protocol that was approved by the institutional review board at Sunnybrook Health Sciences Centre (Toronto, Ontario) and according to the privacy regulations in place at the Institute for Clinical Evaluative Sciences. For the purposes of health services delivery, all transplant centres in Ontario contribute data to Trillium Gift of Life Network (Ontario's organ procurement organization). Trillium has the authority to share this data in a secure manner without patient consent with the Institute for Clinical Evaluative Sciences, where analyses using linked de-identified data are done for the purposes of analysis and quality improvement.

### Healthcare Databases

We identified all deaths (in-hospital and out-of-hospital) in Ontario using the Registered Persons Database (RPDB), which contains vital statistics data on all Ontarians with a valid health card. We used organ procurement billing codes from the Ontario Health Insurance Plan (OHIP), which contains health claims made by physicians. These two databases along with our reference standard database were linked using encrypted unique identifiers. We considered but did not use procedural codes from the Canadian Institutes of Health Information Discharge Abstract Database (CIHI-DAD) in this study, as it is documented that no information on deceased donation is assigned to a patient's file after death (and our own internal work confirmed these codes were not useful for our purposes) [14].

### Algorithms Using Deceased Organ Donor Codes

We compiled a broad list of billing codes from the physician billing database that were relevant to the procurement of organs from a deceased donor (See Table S1 for list of codes). We also considered the billing code for transplant counseling because we thought this code might be billed to most donors. Physician billing claims for the care of each patient are typically submitted by clerical personnel employed by a physician so that the physician can be remunerated for services provided. To establish our final algorithm for detecting deceased organ donors and organ-specific donors, we dropped all billing codes that detected less than one true organ donor, and then combined all remaining codes using the Boolean operator “OR”.

### Definitive Reference Standard

In Ontario, transplant coordinators record in-depth medical information regarding deceased donors at the time of procurement, which are then sent to the province's organ procurement organization (Trillium Gift of Life Network). Using trained research assistants, we abstracted relevant deceased donor characteristics from the medical charts held at Trillium into a separate dataset for the period of January 2006 to March 2011. We considered deceased donors as any individual who donated at least one organ that was subsequently transplanted into a recipient. We also considered deceased donors by organ: heart donor, lung donor, liver donor, kidney donor, pancreas donor, and small intestine donor. Of note, a donor could donate two organs (heart and lung) and would be counted as a heart donor and as a lung donor.

### Statistical Analysis

We calculated the sensitivity, specificity, positive predictive value and negative predictive value for each algorithm compared to the reference standard using standard techniques (where each algorithm was a single code or a combination of codes) [15]. For example, sensitivity was the proportion of deceased organ donors who were successfully identified by the algorithm using billing codes. We calculated 95% confidence intervals for single proportions using the Wilson Score method [16].

### Additional Analyses

#### Restricted cohort

Our initial cohort of all deaths in Ontario was expected to result in a high number of true negatives since most patients who died are not eligible to become deceased organ donors. We also examined our codes in cohorts that excluded individuals who were unlikely to become organ donors in an attempt to increase the proportion of true organ donors in the cohort and improve the positive predictive value. We restricted our cohorts to: (i) hospitalized deaths, (ii) hospitalized deaths associated with at least one code relevant to mechanical ventilation, and (iii) all deaths both in-hospital and out-of-hospital associated with at least one mechanical ventilation code.

#### Comparison of the number of identified vs. expected donors vs. reference standard

The Canadian Organ Replacement Registry (CORR) collected and reported data annually on the number of deceased donors from 10 organ procurement organizations in Canada. They defined a deceased organ donor as any decedent from whom at least one organ was procured and then transplanted into another individual. To determine if the number of donors identified with our final algorithm was similar to expected rates, we compared the number of deceased organ donors in Ontario each year from CORR to the number of deceased organ donors in Ontario identified using our algorithm. This included the validation period (January 1, 2006 to March 31, 2011, and also a pre-validation period January 1, 1991 to December 31, 2005).

## Results

The total number of deaths reported in the Registered Persons Database during the study period (in-hospital and out-of-hospital) was 458,074. During this period, there were also 988 unique deceased organ donors identified using the reference standard, including 848 unique deceased kidney donors, 688 liver donors, 298 lung donors, 234 heart donors, 150 pancreas donors, and 6 small intestine donors. Of these donors, 15 (1.8%) did not have a death date and 6 (0.6%) did not have an associated physician billing code and therefore could not be identified in the administrative data. All 988 unique deceased organ donors were counted in the analysis.

The billing code for transplant counseling performed poorly (sensitivity of 7% and positive predictive value of 11%) and was excluded from further consideration. There were 10 billing codes that successfully identified at least one deceased organ donor (See Table S2). These codes were combined into an algorithm, which identified an organ donor if any one of the 10 codes were present. Overall, this algorithm identified 745 individuals as deceased organ donors. Algorithm sensitivity was 75.4% (95% confidence interval (CI): 72.6%, 78.0%) and the positive predictive value was 77.4% (95% CI: 74.7%, 80.0%) (Table 1). As expected, specificity and negative predictive value were near 100% given the low prevalence of organ donation.

**Table 1:** Validity measures using chart abstraction as the reference standard for the identification of deceased organ donor cases by the best-performing billing coding algorithms.

Donor Type	Sensitivity (95% CI)	Positive Predictive Value (95% CI)	Specificity (95% CI)
Deceased Organ Donor			
Deceased organ donor	75.4% (72.6%, 78.0%)	77.4% (74.7%, 80.0%)	100.0% (100.0%, 100.0%)
Deceased Organ Donor by Specific Organ			
Deceased heart donor	66.7% (60.4%, 72.4%)	36.8% (32.3%, 41.5%)	100.0% (100.0%, 100.0%)
Deceased lung donor	55.7% (50.0%, 61.2%)	52.0% (46.6%, 57.5%)	100.0% (100.0%, 100.0%)
Deceased liver donor	35.6% (32.1%, 39.3%)	66.0% (61.1%, 70.7%)	100.0% (100.0%, 100.0%)
Deceased kidney donor	23.6% (20.9%, 26.6%)	71.2% (65.6%, 76.2%)	100.0% (100.0%, 100.0%)

The codes operated poorly for the detection of organ-specific donation. Algorithms for the identification of heart donors had the best sensitivity (66.7%), followed by lung (55.7%), liver (35.6%), and kidney (23.6%) donors. Algorithms for the identification of kidney donors had the best positive predictive value (71%), followed by liver (66%), lung (52%), and heart (37%) donor algorithms. Physician billing codes were not able to identify any pancreatic and small intestine donors.

### Additional Analyses

Attempts to improve our algorithms by restricting the cohort to deaths that were more likely to be eligible for donation were ineffective. First, we were not able to identify 15% of our reference standard donors when we restricted our cohort to hospitalized deaths. Second, we were not able to improve the positive predictive value when we restricted our cohort to all deaths (in and out-of-hospital) and at least one code relevant to mechanical ventilation at the time of death (results not shown).

**Figure 1:**
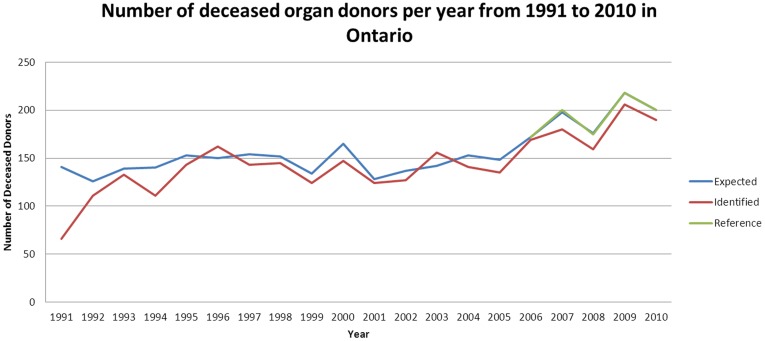
Number of deceased organ donors per year from 1991 to 2010 in Ontario. Expected (blue line) represents the number of deceased organ donors as reported by the Canadian Organ Replacement Registry. Identified (red line) represents the number of deceased organ donors as reported by our physician billing algorithm. Reference (green line) represents the number of deceased organ donors as reported by manual chart reviews from 2006 to 2010.

According to CORR, there were 3126 deceased donors (with at least one organ transplanted) in Ontario during the period of 1991 to 2010 (Figure 1). With our best-performing algorithm (consisting of any of 10 billing codes), we identified 2872 deceased donors during the same period. Our algorithm identified a significantly higher number of heart transplants and much lower rates of kidney transplants and liver transplants (See Figure S1). The number of lung donors did not appear to be significantly different. During the pre-validation period (1991–2005), the billing algorithm identified 474 lung donors compared to an expected 529 donors.

## Discussion

We analyzed all deaths that were registered in administrative health databases in Ontario and identified organ donation with an algorithm using the physician billing databases. We recommend that primary data abstraction preferentially be used to identify deceased organ donors, particularly for the detection of organ-specific donors. When such primary data are not available, the limitations of physician billing claims should be considered whenever they are used. We found that the estimated number of decedents labeled as organ donors each year by our algorithm was similar to that obtained from the national registry. Our algorithm identified deceased organ donors in our province with 75.4% sensitivity and 77.6% positive predictive value compared to the reference standard of actual donations that were recorded in the procurement agency's database and confirmed through manual chart review. This level of sensitivity is similar for algorithms finding other conditions such as deaths (60% sensitivity; 94.8% positive predictive value) in Japanese claims data [17]. When used, methods to correct for misclassification can be considered [18].

The algorithms were largely ineffective at identifying organ-specific donors, highlighting the need for better coding of organ donation in administrative data. For example, the codes were not able to capture any pancreatic and small intestine donors. Conversely, we identified a larger number of deceased heart donors using administrative data compared to the national registry. It is important to note that deceased heart and lung donors share a common code, “M157” (donor heart and lung removal) that could have resulted in a higher number of false positives in our analysis. Other reasons may be that family members consented only for the procurement of lungs and not hearts and concerns about ischemic damage to hearts from donors after cardiac death [19]. Unless administrative data coding is improved, accurate information about organ-specific donations will require alternative strategies, for example manual chart review and data collection.

From our data sources we could not understand why the codes resulted in limited sensitivity. However, some surgeons may receive funding from alternative payment programs and may not always bill for these codes. Further, we spoke to a few billing clerks and found that it may have been possible that surgeons billed the recipients rather the donor for these services. We were surprised that the transplant counseling performed very poorly at identifying deceased organ donors. The purpose of this service is to provide a potential donor's family members with sufficient information and clinical data to enable them to make an informed decision regarding organ donation and transplantation. However, in recent years, organ procurement organizations began employing designated transplant coordinators responsible for obtaining consent for donation from family members. As a result, physicians may play a smaller role in transplant counseling and therefore do not bill for this particular service.

Future research should examine other central elements in the deceased organ donor process. As a priority, algorithms should be developed and validated to identify all deaths eligible for deceased organ donation in large administrative databases. This project would require manual chart abstractions of all those who were deemed eligible and approached for organ donation to serve as the reference standard. However, such project may not be feasible with Ontario's administrative data based on our experience with this study. We will not be able to use any diagnosis or procedural hospital codes that are associated with being an eligible donor because 15% of our reference standard donors were missing when we restricted our cohort to hospitalized deaths. Further, in our pilot work, we were not able to identify any billing code associated with mechanical ventilation that was common among all donors.

Many countries hoping to improve organ donation will require information on their own deceased donors. We recommend that primary data collection (i.e. chart abstraction) be conducted to collect accurate information on deceased donors, particularly organ-specific donors. If primary data collection is too costly or timely, decision-makers may consider the use of physician claims to identify deceased donors while recognizing its limitations. Other alternative approaches to identify deceased organ donors beyond the use of chart abstraction and physician claims include the use of national donor registries. Certain countries may have national donor registries that could be cross-linked to other databases. However, some national registries do not collect or distribute patient identifiers that are needed to link clinical or claims data [20]. An advantage of using administrative data is that it will provide similar information on both those who did and did not become donors (the latter is not recorded in donor registries).

Our study has several strengths. We conducted manual, comprehensive reviews of the medical charts of all deceased donors in our province for the period of interest to create a reference standard. This approach served to minimize the risk of misclassifying organ donors in our reference standard. Our algorithm utilized existing, population-based databases that can be used to study patterns of organ donation across entire regions. Ours is the first study to demonstrate that identifying deceased organ donors using billing algorithms in administrative data has certain limitations. Our experience will help guide the way for future system-level research in Ontario. For example, a recent study used our codes to compare donor rates between transplant hospitals and large general hospitals from 1994 to 2011 [21].

There are several limitations to our study. We used a simple and pragmatic approach to identify organ donors using administrative data, and future research is required to further refine methods for identifying organ-specific donations. Although our algorithm displayed reasonable performance in correctly identifying deceased organ donors, improvements in the performance of the algorithm may be possible using alternative approaches. For example, future work can use machine learning techniques to take information from large administrative databases in an automated fashion to compile the most efficient algorithms [22]. Further, neurological determination of death donors were the most popular donors during our study period. The popularization of donors after cardiac deaths may require different algorithms. In addition, we were not able to identify organs that were procured but not transplanted. Finally, our algorithm used a physician billing database that is unique to Ontario and thus the accuracy of the algorithm may differ in other regions. This limitation applies to all studies examining the validity of case-finding codes in administrative databases [7], [11], [23]–[27]. Similar to other validation studies, the translation of this algorithm to comparable codes in other regions would need to be verified [9]. Our findings are likely of interest to other healthcare systems similar to Ontario where fee-for-service billing is the primary method of physician reimbursement.

In conclusion, our findings reveal that researchers should use primary data abstraction to identify deceased organ donors in large healthcare databases. In healthcare systems where a fee-for-service billing is the primary method of physician reimbursement, researchers may consider using physician claims data to identify deceased organ donors if such data does not exist or is not feasible to collect. However, physician claims may result in missed unidentified cases of organ donation. Further, using physician claims data to identify organ-specific donors (e.g. heart donors) will require improved coding and is not recommended. Our best-performing algorithm for identifying organ donation after death consisted of a combination of heart, lung, liver and kidney procurement billing codes. When primary data are not available, this algorithm can be used in future research to examine trends in deceased organ donation, to evaluate regional disparities in organ donation, and to explore factors that influence its likelihood.

## Supporting Information

Table S1 **List of physician claims codes that were considered in algorithm development.**
(DOCX)

Table S2 **Final algorithm.** An individual was identified as a deceased organ donor in the healthcare databases if any one of these codes was present
(DOCX)

Figure S1 **Number of organ specific donors per year from 1991 to 2010.**
(DOCX)
