# Supplementary material for: Validity of Physician Billing Claims to Identify Deceased Organ Donors in Large Healthcare Databases
Source: PLoS One. 2013 Aug 14;8(8):e70825. doi: 10.1371/journal.pone.0070825 (PMC3743842; doi:10.1371/journal.pone.0070825)
Supplement: Table S1 — List of physician claims codes that were considered in algorithm development. (DOCX) [file pone.0070825.s001.docx]

**Table S1.** List of physician claims codes that were considered in algorithm development

| **OHIP Billing Codes** | |
| --- | --- |
| **Heart procurement from deceased cadaver** | |
| M157 | Donor Heart - Lung removal |
| R870 | Orthotopic cardiac transplantation |
| R872 | Donor cardiectomy |
| R874 | Cardiopulmonary transplantation |
| **Liver procurement from deceased cadaver** | |
| S275 | partial lobectomy (excision greater than 5 cm) |
| S269 | local excision of lesion (less than 5 cm) |
| S267 | Excision – Hepatectomy of three or four liver segments |
| S271 | Excision – Hepatectomy of five or more liver segments |
| S274 | Deceased donor, liver removal |
| S294 | Deceased donor, liver transplant |
| S295 | Repeat liver transplant |
| **Kidney procurement from deceased cadaver** | |
| G347 | Renal perfusion with hypothermia for organ transplantation |
| G348 | Renal preservation with continuous machine perfusion |
| G411 | Nephrological management of donor procurement . |
| S434 | Kidney re-transplant |
| S435 | Kidney transplant |
| S436 | Donor nephrectomy-unilateral orbilateral (to include renal perfusion with hypothermia when rendered by surgeon) |
| **Lung procurement from deceased cadaver** | |
| M155 | Lung transplant (one lung) |
| M156 | Repeat lung transplant (one lung) |
| M157 | Donor Heart - Lung removal |
| **Pancreas procurement from deceased cadaver** | |
| S302 | Donor pancreas removal . |
| S303 | Back-bench pancreas graft preparation |
| S308 | Pancreas transplant |
| **Small intestine procurement** | |
| S196 | Multivisceral transplant – donor |
| S197 | Multivisceral transplant – recipient |
| S201 | Small bowel transplant – donor |
| S202 | Small bowel transplant - recipient |
| **Transplant Counselling** | |
| K014 | Counselling for transplant recipients, donors or families of recipients and donors – 1 or more persons. |
