# Supplementary material for: Validity of Physician Billing Claims to Identify Deceased Organ Donors in Large Healthcare Databases
Source: PLoS One. 2013 Aug 14;8(8):e70825. doi: 10.1371/journal.pone.0070825 (PMC3743842; doi:10.1371/journal.pone.0070825)
Supplement: Table S2 — Final algorithm. An individual was identified as a deceased organ donor in the healthcare databases if any one of these codes was present (DOCX) [file pone.0070825.s002.docx]

**Table S2.** Final algorithm. An individual was identified as a deceased organ donor in the healthcare databases if any one of these codes was present.

| **OHIP Billing Codes** | |
| --- | --- |
| **Heart procurement from deceased cadaver** | |
| M157 | Donor Heart - Lung removal |
| R872 | Donor cardiectomy |
| **Liver procurement from deceased cadaver** | |
| S271 | Excision – Hepatectomy of five or more liver segments |
| S274 | Deceased donor, liver removal |
| S294 | Deceased donor, liver transplant |
| **Kidney procurement from deceased cadaver** | |
| G347 | Renal perfusion with hypothermia for organ transplantation |
| G348 | Renal preservation with continuous machine perfusion |
| G411 | Nephrological management of donor procurement . |
| S435 | Kidney transplant |
| S436 | Donor nephrectomy-unilateral orbilateral (to include renal perfusion with hypothermia when rendered by surgeon) |
| **Lung procurement from deceased cadaver** | |
| M157 | Donor Heart - Lung removal |
