# Supplementary figures and images for: Validity of Physician Billing Claims to Identify Deceased Organ Donors in Large Healthcare Databases
Source: PLoS One. 2013 Aug 14;8(8):e70825. doi: 10.1371/journal.pone.0070825 (PMC3743842; doi:10.1371/journal.pone.0070825)

**Figure S1.** Number of organ specific donors per year from 1991 to 2010

**
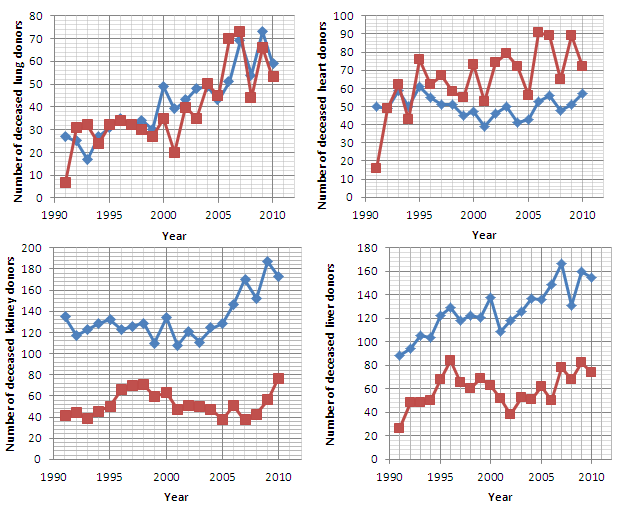
**
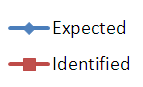

Supplement: Figure S1 — Number of organ specific donors per year from 1991 to 2010. (DOCX) [file pone.0070825.s003.docx]
